# Clinical Outcome of Isolated Cerebellar Stroke—A Prospective Observational Study

**DOI:** 10.3389/fneur.2018.00580

**Published:** 2018-07-17

**Authors:** Alina Nickel, Bastian Cheng, Hans Pinnschmidt, Emine Arpa, Christos Ganos, Christian Gerloff, Götz Thomalla

**Affiliations:** ^1^Department of Neurology, University Medical Center Hamburg-Eppendorf, Hamburg, Germany; ^2^Institute of Medical Biometry and Epidemiology, University Medical Center Hamburg-Eppendorf, Hamburg, Germany; ^3^Department of Neurology, Charité, University of Medicine Berlin, Berlin, Germany

**Keywords:** ataxia, MRI, stroke, cerebellum, MICARS

## Abstract

**Background:** The aim of this prospective study was to investigate clinical deficits of patients with isolated cerebellar stroke applying a dedicated clinical score, the modified International Cooperative Ataxia Rating Scale (MICARS) and identifying factors that influence recovery.

**Methods:** Fifteen patients with acute isolated cerebellar stroke received a standard stroke MRI on the day of admission and were clinically assessed using the mRS, NIHSS and the modified International Cooperative Ataxia Rating Scale (MICARS) on day 1, 3, 7, 30, and 90. A generalized linear model for repeated measures was employed to analyze the effect of stroke lesion location, volume, days after stroke, patient age, and MICARS score at admission on the total MICARS score.

**Results:** Median patient age was 54 years, lesion location in most cases was right (87%) and in the PICA territory (11/15). Median lesion volume was 3.2 ml. Median NIHSS was 1. The median MICARS decreased from on day 1 with 23–4 at day 90. The generalized linear model identified MICARS score at day 1, lesion location, days after admission and the interaction of the last two on the total MICARS score, whereas there was no significant effect of stroke volume or patient age.

**Conclusions:** Isolated cerebellar stroke can present with low NIHSS while more specific scales like the MICARS indicate a severe deficit. Patient age at onset of stroke and lesion volume had no significant effect on recovery from cerebellar symptoms as opposed to severity of symptoms at admission and lesion location.

## Introduction

Acute cerebellar stroke is a relatively rare subtype of acute stroke representing approximately 3% of all ischemic and hemorrhagic strokes (1, 2). Clinical symptoms of cerebellar stroke are manifold and can be subtle so that they are often not recognized at hospital admission (2). Symptoms are frequently underestimated or missed by standard clinical stroke scores such as the National Institutes of Health Stroke Scale (NIHSS). Dedicated clinical scales such as the MICARS are available (3) but not widely used in routine stroke diagnostic and treatment.

Imaging of cerebellar stroke also may be challenging. Whereas small ischemic lesions in the cerebellum are detectable by magnetic resonance diffusion weighed imaging (DWI), identification may be difficult or impossible on computed tomography (CT) (4). Missed diagnosis of cerebellar stroke is not only detrimental to the diagnostic work up of stroke etiology of individual patients but can also lead to serious complications (4).

Studies addressing the clinical course and functional outcome of patients with isolated cerebellar stroke are scarce, and little is known about factors that influence recovery from isolated cerebellar stroke. Therefore, we aimed to investigate the clinical course and prognostic factors of clinical deficits caused by isolated ischemic cerebellar stroke confirmed by Magnetic resonance imaging (MRI) in a prospective study applying a dedicated cerebellar symptom rating scale.

## Materials and methods

### Patients

In a prospective, observational study, patients were recruited from the Stroke Units of two hospitals in Hamburg, Germany, between March 2011 and December 2012. Inclusion criteria were: isolated cerebellar stroke as detected by DWI, MRI within 72 h after stroke onset, age 18 years or older. Patients with stroke lesions involving the brainstem and those with contraindications against MRI were excluded. Written informed consent was obtained from all participants prior to study inclusion. The study was approved by the local ethics committee.

### Clinical assessment

Patients were examined on the day of admission (day 1) and consecutively 3, 7, 30, and 90 days after symptom onset. Clinical assessment included the modified International Cooperative Ataxia Rating Scale (MICARS) (3) (cf. Supplementary Material), Barthel Index (BI), modified Rankin Scale (MRS) and the National Institutes of Health Stroke Scale (NIHSS). NIHSS was only assessed on day 1, whereas the other measures were taken on all time points.

The MICARS is a clinical symptom score dedicated to cerebellar deficits specified in four subcategories: posture and gait disturbances, kinetic functions, speech disorders and oculomotor disorders. The highest possible score, implying highest deficits, is 120 Points (3).

### Imaging

MRI examinations were conducted on a 1.5 T body scanner (Magnetom Symphony/Sonata; Siemens, Erlangen/Germany) up to 72 h after symptom onset. Standardized imaging protocols for the examination of acute stroke patients were used. For this study, data from diffusion-weighted imaging (DWI) were used for further analysis.

### Image processing

Stroke lesion volumes were calculated from seed-based semi-automatic segmentation of apparent diffusion coefficient (ADC) maps applying a threshold of 550 mm/s^2^ using the in-house software ANTONIA, a multi-purpose analysis tool of stroke lesion analysis as described previously (5). In addition, lesion location was visually determined in relation to the corresponding arterial territory using standardized anatomical atlases.

### Statistics

We fitted a generalized linear model for repeated measures to data values of the dependent variable “total MICARS score,” assuming a negative-binomial data distribution and a log-link function (SPSS routine GENLIN). The model was adjusted for patient age, lesion location, lesion volume, MICARS score at baseline examination and time after stroke onset in days. Two-way interaction terms were omitted from the model if they were not significant. Prior to analysis, all continuous independent variables except volume and patient age were log-transformed to achieve normal distribution. The significance level was set to 0.05. All statistical analyses were performed with IBM SPSS 25.0.

## Results

Twenty two patients were initially included in the study. Of these, seven patients discontinued participation. Therefore, 15 patients were finally included in the analysis. Of those, 11 were male (74%). Median age at onset was 54 years (range 31–74). Lesion side was left in 13% (2/15) and right in 87% (13/15) of patients. Cerebellar lesions were located most often in the PICA territory (11/15), followed by SCA (3/15) and combined PICA and AICA territory (1/15). Median ischemic stroke lesion volume was 3.2 ml (range 0.4–25 ml). All patient data is shown for days 1 and 90 after stroke onset (Online Data Supplement).

**Figure 1:**
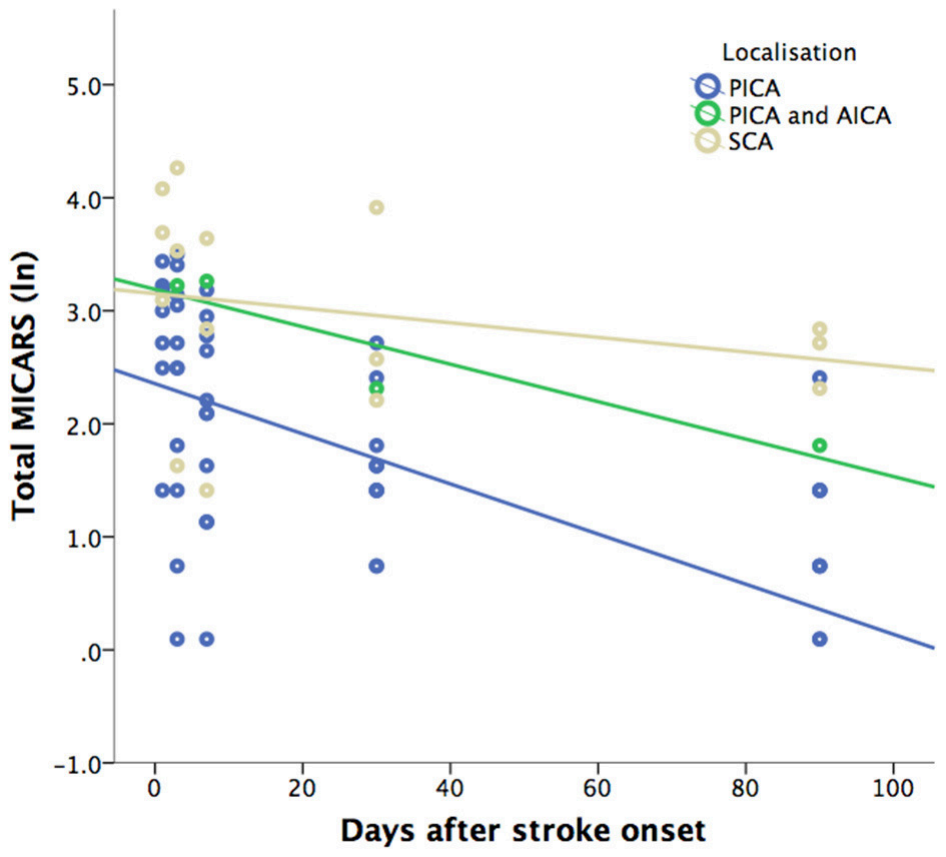
Scatterplot of total MICAR score (plotted as natural logarithm) for all patients during 90 days after stroke onset grouped by lesion localization. Note that MICARS was not assessed for five patients at onset. PICA, posterior inferior cerebellar artery; AICA, anterior inferior cerebellar artery; SCA, superior cerebellar artery; ln, natural logarithm.

Cerebellar symptoms as rated by MICARS were present in all patients with median score of 23 (range 4–59), while NIHSS values were low (median 1; range 0–5). All patients recovered from cerebellar deficits as demonstrated by lower MICARS values at the end of the study (median 4; range 0–17) as reported in Figure 1.

The generalized linear model identified a significant effect on the severity of cerebellar deficits (MICARS) for the following covariates: MICARS score at baseline (Wald Chi = 11.2; *p* < 0.001), lesion location (Wald Chi = 8.4; *p* = 0.015), and days after symptom onset (Wald Chi = 54.2; *p* < 0.001), indicating more severe symptoms in patients with higher baseline MICARS values, lesions in the SCA territory, and a continuous decrease of MICARS values over time. The interaction between lesion localization and days after symptom onset also had a significant effect (Wald Chi = 6.6; *p* = 0.036). There was no significant effect of lesion volume (Wald Chi = 3.0; *p* = 0.08) and age at onset (Wald Chi = 0.2; *p* = 0.7).

## Discussion

Our study has yielded three key findings: First, clinical deficits after isolated cerebellar stroke were subtle and poorly represented by the NIHSS, whereas both the initial clinical deficit as well as clinical recovery were captured validly by MICARS. Second, patients with isolated cerebellar stroke had favorable clinical outcome 3 months after stroke. Lastly, the degree of clinical recovery was mainly influenced by lesion location, while the classical predictors of outcome in supratentorial stroke, i.e., lesion volume and patient age, had no effect on clinical outcome in our sample.

Clinical symptoms of isolated cerebellar lesions are not well represented in conventional clinical stroke scores, and the MICARS has been shown to be superior in the detection and characterization of cerebellar symptoms compared to the NIHSS (6). Confirming these results, in our study some patients suffered from cerebellar symptoms but scored an NIHSS of zero.

Our findings with regards to outcome are in line with previous studies demonstrating that cerebellar stroke generally yields a favorable outcome (7, 8). We demonstrate that in a sample of patients with overall smaller lesion volumes (median of 3.4 ml in our study), clinical outcome was excellent and not modified by the initial size of the ischemic lesion.

In our patients, symptom severity and rate of clinical recovery were significantly influenced by lesion location. Patients with lesions in the PICA territory demonstrated better outcome measured by MICARS compared to patients with lesions affecting cerebellar structures located in the SCA territory and recovered more quickly as demonstrated by the significant interaction between days after stroke onset and lesion location (Figure 1). These findings are in line with a previous study demonstrating that lesions in the SCA territory result in more severe postural impairment and gait ataxia compared to PICA territory infarcts (9).

Our study is limited by the relatively small number of patients. In addition, we are not able to report on the potentially more severe clinical course of patients with larger ischemic lesions due to the relatively small lesion volumes included in our study, and because we excluded patients with additional brainstem infarction which often determines the clinical course.

In conclusion, we present novel and comprehensive data on the longitudinal course of patients with isolated cerebellar infarctions. Patients recovered well during 90 days after stroke onset as measured by a dedicated clinical symptom score (MICARS). Recovery was shown to be dependent from lesion location, with specifically good prognosis in patients with PICA territory stroke. Further studies are needed to examine potential clinical deterioration in patients with larger cerebellar stroke.

## Author contributions

All authors, AN, BC, HP, EA, CGa, CGe, and GT, have substantially contributed to the conception, design, analysis and interpretation of the data as well as to drafting the article and revising it critically. All authors have read and approved the final version of the manuscript.

### Conflict of interest statement

The authors declare that the research was conducted in the absence of any commercial or financial relationships that could be construed as a potential conflict of interest.
