# Supplementary material for: Clinical Outcome of Isolated Cerebellar Stroke—A Prospective Observational Study
Source: Front Neurol. 2018 Jul 17;9:580. doi: 10.3389/fneur.2018.00580 (PMC6056646; doi:10.3389/fneur.2018.00580)
Supplement: Supplementary file 1 [file Table_1.DOCX]

| **ID** | **Age** | **Gender** | **Lesion Localisation** | **Lesion Volume [ml]** | **MICARS** | | **mRS** | | **BI** | | **NIHSS** |
| --- | --- | --- | --- | --- | --- | --- | --- | --- | --- | --- | --- |
|  |  |  |  |  | Day 1 | Day 90 | Day 1 | Day 90 | Day 1 | Day 90 | Day 1 |
| 1 | 46 | F | right PICA | 16,23 | 24 | 0 | 4 | 0 | 50 | 100 | 1 |
| 2 | 57 | M | left PICA | 1,1 | 12 | 2 | 0 | 0 | 100 | 100 | 2 |
| 3 | 51 | M | right PICA | 18,3 | 20 | 4 | 0 | 0 | 100 | 100 | 1 |
| 4 | 36 | F | right PICA | 0,4 | 15 | 2 | 2 | 0 | 75 | 100 | 0 |
| 5 | 74 | M | right SCA | 2,25 | 22 | 10 | 2 | 0 | 85 | 100 | 2 |
| 6 | 70 | F | right PICA | 1,26 | 31 | 4 | 2 | 0 | 60 | 100 | 5 |
| 7 | 54 | M | right PICA | 2,4 | n/a | 0 | n/a | 0 | n/a | 100 | 2 |
| 8 | 71 | M | right SCA | 13,5 | 59 | 15 | 4 | 1 | 45 | 100 | 5 |
| 9 | 35 | M | right SCA | 2 | 40 | 17 | 4 | 0 | 30 | 100 | 1 |
| 10 | 68 | M | left PICA/AICA | 1,7 | n/a | 6 | n/a | 0 | n/a | 100 | 1 |
| 11 | 73 | M | right PICA | 15,9 | 25 | 1 | 0 | 0 | 100 | 100 | 2 |
| 12 | 54 | M | right PICA | 3,2 | n/a | 2 | n/a | 0 | 100 | 100 | 0 |
| 13 | 70 | M | right PICA | 25 | n/a | 11 | 2 | 0 | 100 | 100 | 1 |
| 14 | 47 | M | right PICA | 15,6 | 4 | 4 | 0 | 0 | 100 | 100 | 0 |
| 15 | 31 | F | right PICA | 18,8 | n/a | 1 | n/a | 0 | 80 | 100 | 0 |

Table 1: Demographic and clinical data of all patients included in the study. Abbreviations: MICARS: total modified International Cooperative Ataxia Rating Scale on day 1 and day 90, MRS: modified Rankin Scale on day 1 and day 90, BI: Barthel Index on day 1 and day 90, NIHSS: National Institutes of Health Stroke Scale, F: female, M: male, n/a: not applicable, PICA: posterior inferior cerebellar artery, AICA: anterior inferior cerebellar artery, SCA: superior cerebellar artery
