# Supplementary material for: Clinical Outcome of Isolated Cerebellar Stroke—A Prospective Observational Study
Source: Front Neurol. 2018 Jul 17;9:580. doi: 10.3389/fneur.2018.00580 (PMC6056646; doi:10.3389/fneur.2018.00580)
Supplement: Supplementary file 2 [file Table_2.DOCX]

**Modified International Cooperative Ataxia Rating Scale (MICARS)**

**from Schmahmann et al. (2009)(1), Tab. 1**

| I. Posture and gait disturbances |
| --- |
| 1.Walking capacities |
| 0:Normal |
| 1:Almost normal naturally, but unable to walk with feet in tandem position |
| 2: Walking without support, but clearly abnormal and irregular |
| 3: Walking without support but with considerable staggering; difficulties in half turn |
| 4: Walking with autonomous support no longer possible; the patient uses the episodic support of the wall for 10-meter test |
| 5: Walking only possible with one stick |
| 6: Walking only possible with two special sticks or with a Stroller |
| 7: Walking only with accompanying person |
| 8: Walking impossible, even with accompanying person (wheelchair) |
| 2. Gait speed |
| 0: Normal |
| 1: Slightly reduced |
| 2: Markedly reduced |
| 3: Extremely Slow |
| 4: Walking with autonomous support no longer possible |
| 3. Standing capacities, eyes open |
| 0: Normal: able to stand on one foot more than 10 seconds |
| 1: Able to stand with feet together, but no longer able to stand on one foot more than 10 seconds |
| 2: Able to stand with feet together, but no longer able to stand with feet in tandem position |
| 3: No longer able to stand with feet together, but able to stand in natural position without support, with no or moderate sway |
| 4: Standing in natural position without support, with considerable sway and considerable corrections |
| 5: Unable to stand in natural position without strong support of one arm |
| 6: Unable to stand at all, even with strong support of two arms |
| 4. Spread of feet in natural position without support, Eyes open |
| 0: Normal (<10 cm) |
| 1: Slightly enlarged (>10 cm) |
| 2: Clearly enlarged (25 cm < spread < 35 cm) |
| 3: Severely enlarged (>35 cm) |
| 4: Standing in natural position impossible |
| 5. Body sway with feet together, eyes open |
| 0: Normal (<10 cm) |
| 1: Oscillations |
| 2: Moderate oscillations (<10 cm at the level of head) |
| 3: Severe oscillations (>10 cm at the level of head), threatening the upright position |
| 4: Immediate falling |
| 6. Body sway with feet together, eyes closed |
| 0: Normal (<10 cm) |
| 1: Slight oscillations |
| 2: Moderate oscillations (<10 cm at the level of head) |
| 3: Severe oscillations (>10 cm at the level of head), threatening the upright position |
| 4: Immediate falling |
| 7. Quality of sitting position |
| 0: Normal |
| 1: With slight oscillations of the trunk |
| 2: With moderate oscillations of the trunk and legs |
| 3: With severe dysequilibirium |
| 4: Impossible |
| II. Kinetic functions |
| 8. Knee-tibia test (decomposition of movement and intention tremor) (Left and Right scored) |
| 0: Normal |
| 1: Lowering of heel in continuous axis, but the movement is decomposed in several phases, without real jerks, or abnormally slow |
| 2: Lowering jerkily in the axis |
| 3: Lowering jerkily with lateral movements |
| 4: Lowering jerkily with extremely long lateral movements or test impossible |
| 9. Action tremor in the heel-to-knee test (Left and Right scored) |
| 0: Normal |
| 1: Tremor stopping immediately when the heel reaches the knee |
| 2: Tremor stopping <10 seconds after reaching the knee |
| 3: Tremor continuing >10 seconds after reaching knee |
| 4: Uninterrupted tremor or test impossible |
| **10. Decomposition of leg movement (Left and Right scored)** |
| **0: Normal** |
| **1: Corners or edges on the circle** |
| **2: Markedly decomposed attempts at circle** |
| **11. Decomposition of leg tapping (Left and Right scored)** |
| **0: Normal** |
| **1: Slightly slow and irregular** |
| **2: Clearly slow and irregular** |
| 12. Finger-to-nose test: decomposition and dysmetria (Left and Right scored) |
| 0: Normal |
| 1: Oscillating movement without decomposition of the movement |
| 2: Segmented movement in 2 phases and/or moderate dysmetria in reaching nose |
| 3: Segmented movement in more than 2 phases and/or considerable dysmetria in reaching nose |
| 4: Dysmetria preventing the patient from reaching nose. |
| 13. Finger-to-nose test: intention tremor of the finger (Left and Right scored) |
| 0: Normal |
| 1: Simple swerve of the movement |
| 2: Moderate tremor with estimated amplitude <10 cm |
| 3: Tremor with estimated amplitude between 10 cm and 40 cm. |
| 4: Severe tremor with estimated amplitude >40 cm |
| 14. Finger-finger test (action, tremor and/or instability) (Left and Right scored) |
| 0: Normal |
| 1: Mild instability |
| 2: Moderate oscillations of finger with estimated amplitude <10 cm |
| 3: Considerable oscillations of finger with estimated amplitude between 10 and 40 cm |
| 4: Jerky movements >40 cm of amplitude |
| 15. Pronation-supination alternating movements (Left and Right scored) |
| 0: Normal |
| 1: Slightly irregular and slowed |
| 2: Clearly irregular, and slowed movement, but without elbow sway |
| 3: Extremely irregular, and slowed, but with sway of the elbow |
| 4: Movement completely disorganized or impossible |
| **16. Rebound of the arms (Left and Right scored)** |
| **0: None** |
| **1: Less than 10 cm** |
| **2: Greater than 10 cm** |
| **17. Overshoot of the arms (Left and Right scored)** |
| **0: None** |
| **1: Less than 10 cm** |
| **2: Greater than 10 cm** |
| 18. Drawing of Archimedes’ spiral on a predrawn pattern |
| 0: Normal |
| 1: Impairment and decomposition, the line quitting the pattern slightly, but without hypermetric swerve |
| 2: Line completely out of the pattern with recrossings and/or hypermetric swerves |
| 3: Major disturbances due to hypermetria and decomposition |
| 4: Drawing completely disorganized or impossible |
| 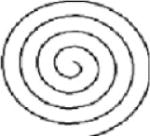 |
| III. Speech disorders |
| 19. Dysarthria: fluency of speech |
| 0: Normal |
| 1: Mild modification of fluency |
| 2: Moderate modification of fluency |
| 3: Considerable slow and dysarthric speech |
| 4: No speech |
| 20. Dysarthria: Clarity of speech |
| 0: Normal |
| 1: Suggestion of slurring |
| 2: Definite slurring, most words understandable |
| 3: Severe slurring, speech not understandable |
| 4: No speech |
| **21. Dysarthria: Alternating syllables** |
| **0: Normal** |
| **1: Slightly irregular** |
| **2: Clearly irregular, dysrhythmic and slurred** |
| IV. Oculomotor disorders |
| **22. Abnormal eye movements at rest** |
| **0: Absent** |
| **1: Present** |
| 23. Gaze-evoked nystagmus |
| 0: Normal |
| 1: Transient |
| 2: Persistent but moderate |
| 3: Persistent and severe |
| 24. Abnormalities of the ocular pursuit |
| 0: Normal |
| 1: Slightly saccadic |
| 2: Clearly saccadic |
| 25. Dysmetria of the saccade |
| 0: Absent |
| 1: Bilateral clear overshoot or undershoot of the saccade |
| **26. Saccadic intrusions into vestibulo-ocular reflex cancellation** |
| **0: Absent** |
| **1: Present** |

Reference:

1. Schmahmann JD, Gardner R, MacMore J, Vangel MG. Development of a brief ataxia rating scale (BARS) based on a modified form of the ICARS. Mov Disord. 2009;24(12):1820–8.
